# Epidemic Cholera in Butler Co. Ohio

**Published:** 1834

**Authors:** Cyrus Falconer


XV.—Epidemic Cholera in Butler County, Ohio. By Dr. Cyrus Falconer.

The progress of the cholera in this country, within the present year, is strongly illustrative of its erratic and implacable character. It appeared first about the 10th of July, and selected for the theatre of its ravages, a section of country heretofore distinguished for its almost unvaried salubrity. The infected district, *i. e.* the district first attacked, and where the disease spent the violence of its assault, embraces a portion of two townships, (Morgan and Ross,) in the southwest angle of the county. "*Paddy's Run*," a small creek, meanders through the centre of the region, bearing the commingled tribute of the adjoining hills to the Miami. *Indian Creek*, a stream somewhat large, flows along its border, and enters the Miami higher up.

The first case is believed to have been a gentleman who a few days previous to his attack had visited Cincinnati, where cholera was then existing; but he recovered, and the nature of his attack is by some held doubtful. The next case was his son, a lad who had accompanied him—he died, and from that period attacks rapidly multiplied. From its commencement to the acme of severity, the march of the disease was frightfully rapid; its continuance, about three weeks—the last week of this time, however, introduced as is, I believe, customary, a milder character and more manageable symptoms.

The mortality in proportion to the number of cases, has been unusually great; indeed, during the first part of the period referred to, the certainty with which death followed an attack was equalled only by its rapidity. This may be attributed, in some measure, at least, to two causes—the excessive panic incident to the unexpected appearance of the pestilence amongst a people uninformed of its characteristics, and consequently vividly impressed with fears of its contagion—and the want of intelligent physicians. One of the first victims was a physician; another medical gentleman was sick during the whole cour^e of the disease, and before physicians from a distance could possibly see a patient, the time to act successfully, had passed forever.

In nearly every case, so far as I have learned, there was a stage of premonition of from *one* to *several* days, and when attended to in this stage the disease was medicable. From the circumstances above related, it will be seen, that in a majority of cases, not much *treatment* was afforded. *Calomel* is the only remedy which appears to have been used with efficacy, and in those families where a prompt and free resort to it was made, it was successful. *Opium*, *ether*, and other antispasmodics were used in some cases, and the *external stimulant* plan generally tried. *Venesection* was, I believe, in no case practised. The *steam system* was tried by a portion of the credulous, and shortened almost invariably, "the road to dusty death."

The first appearance of cholera was in the midst of a rain of several days' duration, and its greatest mortality in a week of remarkably warm weather which succeeded it. I am not aware that any meterological phenomena, or unusual atmospherical appearances were observed immediately previous to, or during the existence of, the disease.

The distance from the Miami to Oxford, (the extreme limits of the infected district in this county,) is about 15 miles. The soil and its productions within this space, resemble those of the Miami valley generally, and the surface of the country presents a more than the usual variety of broken boldness—of hill and valley. The streams are rapid; marshes unknown; and what is certainly remarkable, the points where the destroyer has left the most fatal evidences of his presence, are amongst the most elevated in the district. The miniature vallies bordering the several streams in the route of the cholera were, for the most part, avoided, or lightly touched, while the adjoining highlands suffered severely. One village, (Venice,) in the midst of a level plain, had but 2 or 3 cases; another, (Millville,) a part of which occupies a low and level situation on the edge of Indian Creek, contained but few cases, although a range of hills in its immediate vicinity suffered dreadfully. I am unable to afford any clue to the causes which induced this extraordinary location—they surely will not be found in any theory heretofore promulged, of the origin of cholera, save, perhaps, the *animalcular*.

The disease was announced first within a few miles of the Miami; as it subsided here, it advanced northerly, touching but slightly in its course until it alighted upon Oxford. The situation of this village is too well known to need description; it is enough to say here, that its high and healthful location resembles those of the other places attacked in this county. A glance at the *profile* of the district travelled over by cholera, proves that its advance was south to north, and that it moved in a *direct line* leading from Cincinnati to Oxford. There were a few cases on the east side of the Miami, and in Hamilton county; but I believe that every case occurred within a space bounded by two parallel lines, say 3 miles asunder, leading from Cincinnati to Oxford. Such are the facts—I leave to others their explanation.

The district of country of which I have been speaking, would equal in extent, perhaps, a township, and contains about 1200 inhabitants. The following synopsis exhibiting the number of *fatal* cases, with the proportion of ages and sexes, was compiled from sources which may be relied on for general accuracy. It has been computed, that death resulted in threefourths of all the attacks.—

| Males. | | Females. | |
|---|---|---|---|
| *Deaths.* | *Ages.* | *Deaths.* | *Ages.* |
| 6 | 15 and under | 7 | 13 and under |
| 4 | 15 to 20 | 3 | 15 to 20 |
| 5 | 20 " 30 | 5 | 20 " 30 |
| 6 | 30 " 40 | 4 | 30 " 40 |
| 5 | 40 " 50 | 5 | 40 " 50 |
| 4 | 50 " 60 | 6 | 50 " 60 |
| 8 | over 60 | 4 | over 60 |

Total 72.—There is an unusual number I think of aged persons.

